# Isolation of Bacteria with Antifungal Activity against the Phytopathogenic Fungi *Stenocarpella maydis* and *Stenocarpella macrospora*

**DOI:** 10.3390/ijms12095522

**Published:** 2011-08-29

**Authors:** Iván Petatán-Sagahón, Miguel Angel Anducho-Reyes, Hilda Victoria Silva-Rojas, Ainhoa Arana-Cuenca, Alejandro Tellez-Jurado, Isabel Oyuki Cárdenas-Álvarez, Yuridia Mercado-Flores

**Affiliations:** 1 Universidad Autónoma de Chapingo, Km. 38.5 Carretera México-Texcoco. Chapingo. Estado de México, CP 56230, Mexico; E-Mail: ing_agrozoo19@hotmail.com; 2 Universidad Politécnica de Pachuca, Km. 20 Carretera Pachuca Cd. Sahagún. Rancho Luna, Ex-Hacienda de Sta. Barbara, Municipio de Zempoala, Hidalgo, CP 42184, Mexico; E-Mails: anducho@hotmail.com (M.A.A.-R.); ainhoa@upp.edu.mx (A.A.-C.); tejual@hotmail.com (A.T.-J.); lluviaveraln@hotmail.com (I.O.C.-A.); 3 Colegio de Postgraduados, Producción de Semillas, Km. 36.5 Carretera México-Texcoco. Edo. de México, CP 56230, Mexico; E-Mail: hsilva@colpos.mx

**Keywords:** *Stenocarpella*, biological control, *Bacillus*, *Pseudomonas*, *Pantoea*

## Abstract

*Stenocarpella maydis* and *Stenocarpella macrospora* are the causal agents of ear rot in corn, which is one of the most destructive diseases in this crop worldwide. These fungi are important mycotoxin producers that cause different pathologies in farmed animals and represent an important risk for humans. In this work, 160 strains were isolated from soil of corn crops of which 10 showed antifungal activity against these phytopathogens, which, were identified as: *Bacillus subtilis*, *Pseudomonas* spp., *Pseudomonas fluorescens*, and *Pantoea agglomerans* by sequencing of 16S rRNA gene and the phylogenetic analysis. From cultures of each strain, extracellular filtrates were obtained and assayed to determine antifungal activity. The best filtrates were obtained in the stationary phase of *B. subtilis* cultures that were stable to the temperature and extreme pH values; in addition they did not show a cytotoxicity effect against brine shrimp and inhibited germination of conidia. The bacteria described in this work have the potential to be used in the control of white ear rot disease.

## 1. Introduction

The white ear rot of corn disease is caused by the fungi *Stenocarpella maydis* and *S. macrospora* and is one of the most destructive for corn crops around the world. The symptoms are manifested many weeks after infection, affecting mainly the root, stalk, and cobs that later show a white cottony fungal growth. It is also possible to observe the presence of pycnidia, which is the source for the spreading of the disease. When the symptoms are manifested in the stalk, the internodes show a dark brown color; in this case, the plants become weak and are easily broken by rain and strong winds. The life cycles of both fungi are similar; the difference is that *S. maydis* is present in cooler regions because conidia lose their viability at high temperatures and by exposure to sunlight [1,2]. On the other hand, these fungi are important as mycotoxin producers in stored grains. *S. macrospora* produces diplodiol, a mycotoxin that causes death in chickens and chicks [3]. While *S. maydis* synthesizes diploidiatoxin, the causal agent of diplodiasis, a neuromycotoxicosis, is characterized by neurological alterations such as ataxy, paralysis, and hepatic damage in cattle fed with the infected corn. This toxic effect has been observed in other farm and laboratory animals [4–7].

To control the existence of ear rot in corn hybrids with a high level of resistance to the fungi, the best management method is crop rotation; however, two or three years are needed to reduce the fungal inoculum to acceptable levels [8]. Another alternative is the application of synthetic fungicides; however, the use of these compounds has been reduced due to their high toxicity. In this case, biological control is an attractive option as a component of an integrated disease management scheme given the reduction in the use of chemical compounds [9].

Although different microorganisms can be used as biological control agents, important evidence exists regarding the role of antibiotic production by bacteria isolated from the soil, such as suppressors and inhibitors in the development of phytopathogens [10]. Biological control of *S. maydis* and *S. macrospora* has been achieved at the experimental level with different strains of actinomycetes with the potential to become a tool for the reduction of disease [11,12].

The utilization of other types of bacteria to control these fungi has not been reported yet. In this work, we provide information on the isolation and identification of bacteria isolated from the rhizospheric soil of corn crops with antagonist activity against *S. maydis* and *S. macrospora* with the potential to be used in the biological control of these fungi.

## 2. Results and Discussion

### 2.1. Results

**Figure 1:**
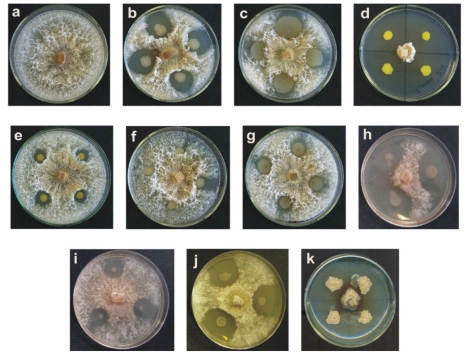
Antifungal activity against *S. maydis* of the bacteria isolated from corn crop soils. (**a**) *S. maydis* control; (**b**) strain 11; (**c**) strain 13; (**d**) strain 16; (**e**) strain 19; (**f**) strain 21; (**g**) strain 35; (**h**) strain 55; (**i**) strain 135; (**j**) strain 156; and (**k**) strain 160. The fungus was inoculated into PDA medium.

**Figure 2:**
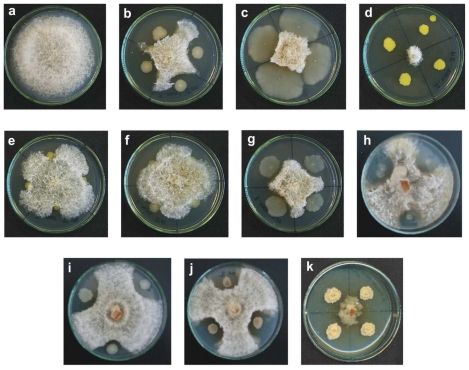
Antifungal activity against *S. macrospora* of the bacteria isolated from corn crop soils. (**a**) *S. macrospora* control; (**b**) strain 11; (**c**) strain 13; (**d)** strain 16; (**e**) strain 19; (**f**) strain 21; (**g**) strain 35; (**h**) strain 55; (**i**) strain 135; (**j**) strain 156; and (**k**) strain 160. The fungus was inoculated into PDA medium.

A total of 160 bacterial strains were isolated from soil samples, of which ten showed antifungal activity against *S. maydis* and *S. macrospora* (Figures 1 and 2, respectively). The strains 13, 35, 55, 135, and 160 were Gram positive and the strains 11, 16, 19, 21, and 156 were Gram negative.

The identity of the isolated bacteria with antifungal activity was determined by amplification and sequencing of the 16S rRNA. The edited sequences were deposited in GeneBank, under accession numbers GU220577 (11), GU220584 (13), GU220578 (16), GU220579 (19), GU220576 (21), GU220581 (35), GU220580 (55), GU220582 (135), GU220575 (156) and GU220583 (160).

**Figure 3:**
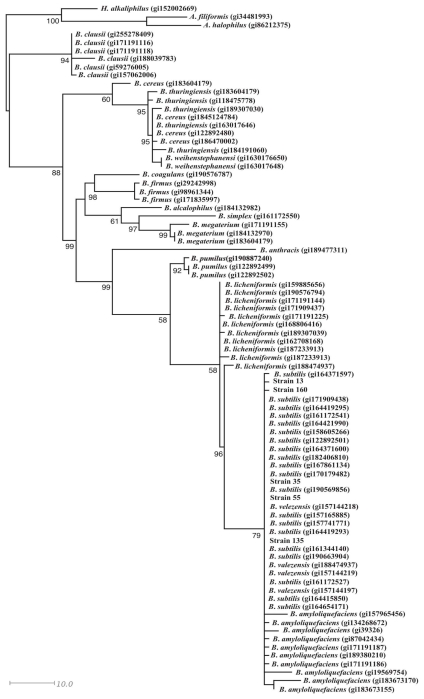
Phylogenetic analysis of the nucleic acid sequences of 16S rRNA of the Gram positive bacteria with antifungal activity.

**Figure 4:**
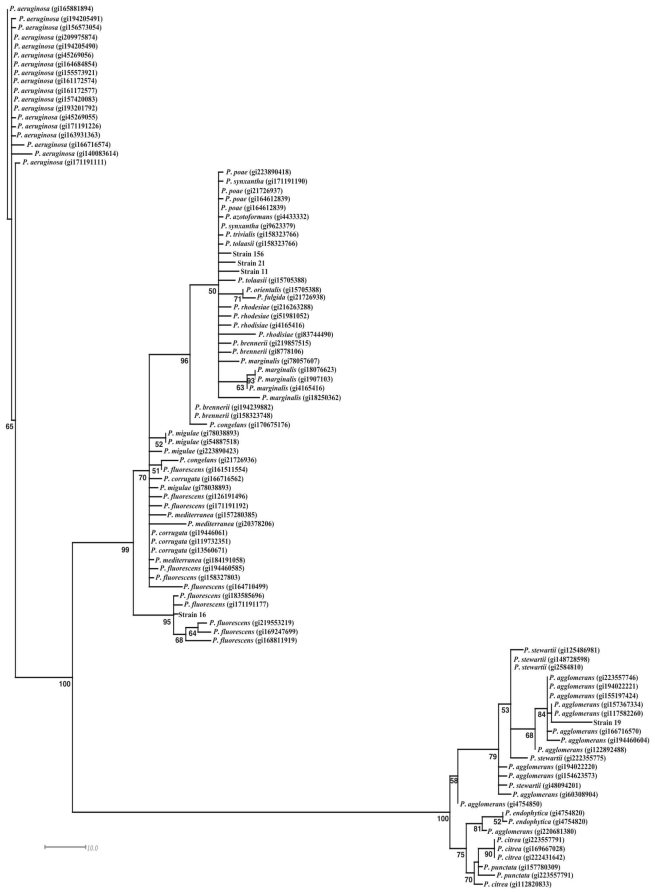
Phylogenetic analysis of the nucleic acid sequences of 16S rRNA of the Gram negative bacteria with antifungal activity.

The phylogenetic analysis showed that the Gram positive strains 13, 35, 55, 135, and 160 were related with *Bacillus subtilis* (Figure 3). Most of the Gram negative strains were related with *Pseudomonas* genera, specifically strains 156, 21, and 11. The bacterial 16 was grouped with *Pseudomonas fluorescens*, whereas the 19 strain was grouped with *Pantoea agglomerans* (Figure 4).

After determining the growth curve of each isolated bacterial strain with antifungal activity, different extracellular filtrates were obtained, as described in Materials and Methods, in this case the sampling was at 8, 16 and 32 h for the Gram negative bacteria and for the Gram positive bacteria was at 12, 24 and 50 h, to correspond at logarithmic, stationary early and stationary late phases respectively. The filtrates obtained from Gram negative bacteria showed a low inhibition or did not inhibit fungal development. However, the samples obtained in all growth phases from the *Pseudomonas* spp. 11 inhibited up to 70% of both fungi and the filtrates obtained in logarithmic phase from the *P. fluorescens* 16 inhibited 54% the growth of *S. maydis*. In general, the filtrates obtained from all strains of *B. subtilis* inhibited considerably the growth of both fungi. Most of the samples with high activity were obtained in the stationary phase (Table 1).

The extracellular filtrates with the best antifungal activity were selected to measure their stability at different conditions of temperature and pH. From *Pseudomonas* spp. 11 and *P. fluorescens* 16, the filtrates were obtained from a culture of 26 and 16 h that corresponded to the stationary and logarithmic phase, respectively. In contrast, in the case of *B. subtilis* 55, the filtrates were obtained from early stationary phase (22 h), and the filtrates selected from *B. subtilis* 13, 35, 135, and 160 strains were those obtained at 36, 35, 38, and 48 h respectively (late stationary phase).

**Figure 5:**
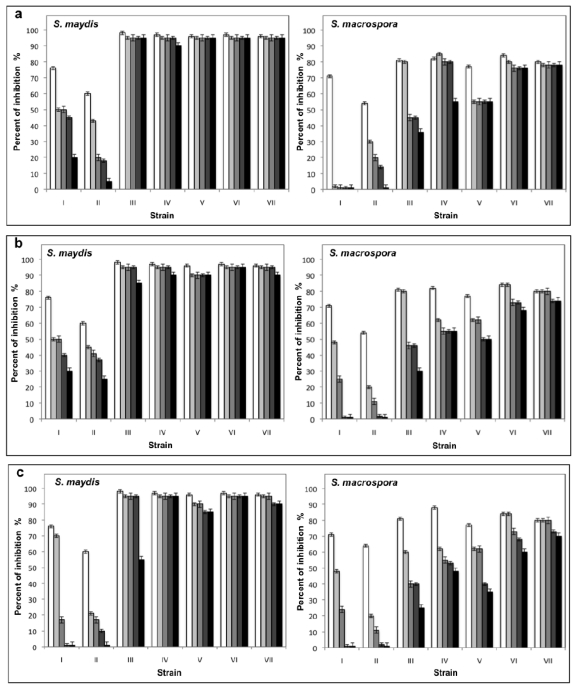
Effects of the pH on the stability of extracellular filtrates with antifungal activity obtained from (I) *Pseudomonas* spp. 11, (II) *P. fluorescens* 16, (III) *B. subtilis* 13, (IV) *B. subtilis* 35, (V) *B. subtilis* 55, (VI) *B. subtilis* 135 and (VII) *B. subtilis* 160. The filtrates were incubated during □ 0, 
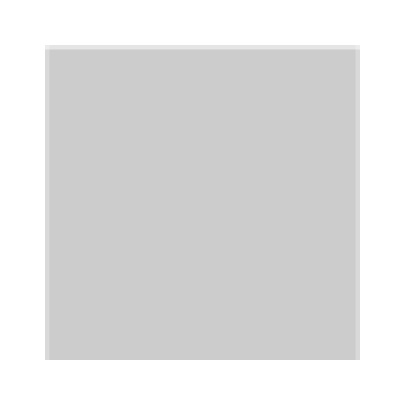
 1, 
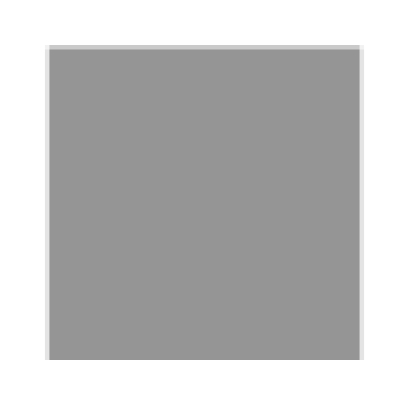
 7, 
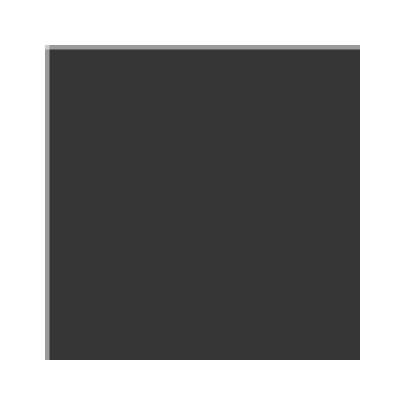
 15, and ■ 30 days in the presence of 0.1 M citrate buffer, pH 3.6 (**a**), 0.1 M phosphate buffer, pH 7 (**b**) and 0.1 M Tris-HCl buffer, pH 10 (**c**), and assayed against *S. maydis* and *S. macrospora* in PDA.

**Figure 6:**
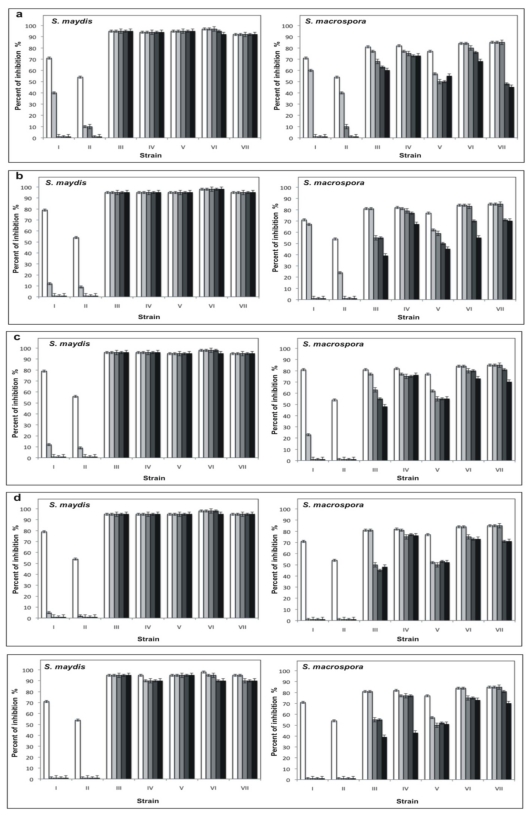
Effect of temperature on the stability of extracellular filtrates with antifungal activity obtained from (I) *Pseudomonas* spp. 11, (II) *P. fluorescens* 16, (III) *B. subtilis* 13, (IV) *B. subtilis* 35, (V) *B. subtilis* 55, (VI) *B. subtilis* 135 and (VII) *B. subtilis* 160. The filtrates were incubated during □ 0, 
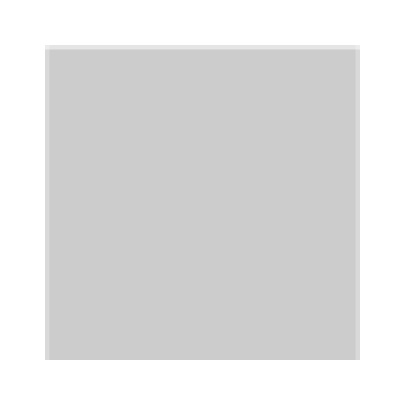
 1, 
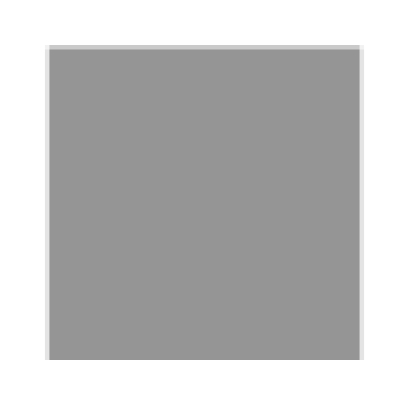
 7, 
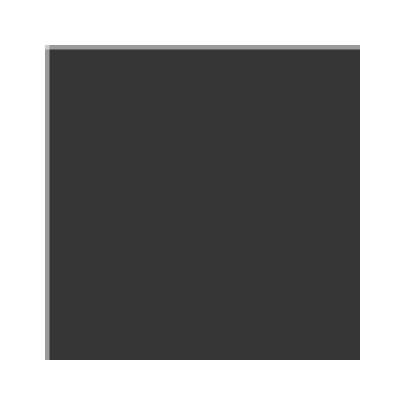
 15 and ■ 30 days at −20 °C (**a**); 4 °C (**b**); 20 °C (**c**); 37 °C (**d**); and 50 °C (**e**) and assayed on *S. maydis* and *S. macrospora* in PDA.

**Figure 7:**
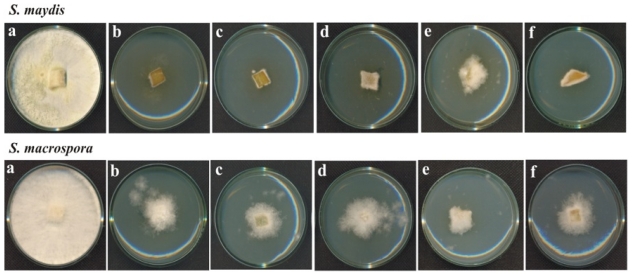
Antifungal activity of the extracellular filtrates sterilized in autoclave. (**a**) Control; (**b**) *B. subtilis* 13; (**c**) *B. subtilis* 35; (**d**) *B. subtilis* 55; (**e**) *B. subtilis* 135; and (**f**) *B. subtilis* 160. The filtrates were obtained as described in the Material and Methods. For the control, the fungi were inoculated in PDA with sterile distilled water.

The filtrates obtained from *Pseudomonas* spp. 11 and *P. fluorescens* 16 did not show stability at the different temperatures and pH conditions assayed, in contrast with the filtrates from *B. subtilis*, which maintained their antifungal activity even after being incubated for 30 days at different temperatures and pH values (Figures 5 and 6). Furthermore, five filtrates obtained from the *B. subtilis* 13, 5, 55, 135, and 160 strains showed stability to sterilization (Figure 7). These thermostable extracts did not show cytotoxicity against brine shrimp. In general, all extracts showed higher antifungal activity on *S. maydis* than on *S. macrospora.*

**Figure 8:**
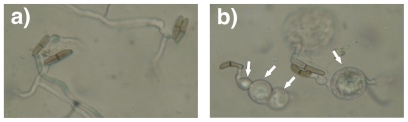
Effect of the thermostable filtrates obtained from *B. subtilis* 160 against the conidia germination of *S. maydis*. (**a**) Normal conidia germination without presence of thermostable filtrate; (**b**) Abnormal conidia germination in presence of thermostable filtrate, the arrows indicates the formation of swollen cells like chlamydospores. Unstained preparations (optical microscopy at 40×).

When the same fltrates were inoculated in plates of YEPD with a conidia suspension of *S. maydis* and *S. macrospora,* no colony development were observed. However, when the plates were observed under the microscope they exhibited abnormal germination tube and no hyphae grew from the swollen cells, which are like chlamydospores and not were present in cultures without extracts (Figure 8).

### 2.2. Discussion

The use of biological control in the management of agriculture pests and diseases is an effective alternative to the use of pesticides, which are often accumulated in plants and are lethal to beneficial organisms present in the soil [13]. Nowadays, the development of new environmentally friendly methodologies, are based on the search for new genetic, chemical, and biological sources that are effective for phytosanitary issues [14]. Although different microorganisms have been utilized as control agents, antibiotics produced by bacteria isolated from the soil contribute to the suppression or inhibition of the growth of phytopathogens [10]. In the present work, the isolation of bacteria from soil of corn crops was performed with the idea of using these strains as biological agents in the integrated management of ear rot caused by *S. maydis* and *S. macrospora.*

Of the 160 bacteria isolated, only 10 showed antifungal activity over the studied fungi through diffusible substances in agar, of which some Gram-negative bacteria showed a higher inhibitory effect than the Gram-positive strains. However, the extracellular filtrates obtained from these same microorganisms presented low antifungal activity. Numerous Gram-negative bacteria have been used in the control of fungal plant pathogens. These bacteria exert their antifungal activity through the production of extracellular lytic enzymes, siderophores, salicylic acid, antibiotics, and volatile metabolites, such as hydrogen cyanide [15–20]. It is possible that some Gram-negative bacteria isolated in this work produce antifungal volatile compounds or enzymatic activities that are lost in the process of obtaining the filtrates. On the other hand, it can be assumed that the presence of bacteria is necessary for the substances responsible of the inhibition, and could be associated with the bacterial envelopes.

Molecular identification showed that most of the Gram-negative bacteria are included in the *Pseudomonas* genus (11, 16, 21, and 156 strains), specifically the 16 strain was grouped in the cluster of *P. fluorescens*. Numerous species of this genus are used as biological control agents of soil pathogens for their ability to colonize plant roots. Some of them can increase plant growth due to a property that inhibits the development of plant pathogenic microorganisms [15,16,20–23]. At present, there are many products containing *Pseudomonas* with moderate to excellent efficacy that are used for plant disease control [24]. Recently, the genomic analysis of this bacterial group has enabled the development of tools for the effective management of biological control agents in the field. However, the inoculation of native strains is important to exploit the beneficial properties of these microorganisms in agriculture because these microorganism are best adapted to conditions in the place when the phytosanitary problem is present [25].

Strain 19 was identified as *P. agglomerans*. This microorganism is used as a biological control agent for the bacterial pathogens *P. syringae* pv. *syringae* and *Erwinia amylovora* causal agents of the basal kernel blight of barley and fire blight in orchards, respectively [26,27]; however, their main use is in post-harvest biological control of fungi in fruits and citrus, inhibiting the development of *Penicillium expansum*, *Botritis cinereae*, *Rhizopus stolonifer*, *Penicillium digitatum*, and *Monilinia laxa*, and therefore, could be an alternative to chemical fungicides [28–34].

The most active filtrates were obtained from the Gram-positive bacteria that were identified as *B. subtilis*. The samples that presented higher inhibition of the fungi under study were obtained in the stationary phase of growth, indicating that the compounds are secondary metabolites. Within the Gram-positive bacteria used as biological control agents, some species of the *Bacillus* genus (*B. subtilis*, *B. licheniformis*, *B. pumilus*, *B. amyloliquefaciens*, and *B. cereus*) inhibit the development of different fungal plant pathogens due to their capacity to produce a broad variety of antibiotics [35–37].

The filtrates obtained from the five strains of *B. subtilis* (13, 35, 55, 135, and 160) maintained their antifungal activity even after being autoclaved, besides being stable at different pH values. Some species of *Bacillus* produce antibiotics with a broad spectrum of action besides being stable to high temperatures and different pH conditions. These compounds are cyclic lipopeptides with an amphipathic character that are divided into three groups: surfactins, iturins, and fengicins, which are secondary metabolites [13,35]. It is possible that the antifungal activities found in the five strains of *B. subtilis* correspond or are similar to these compounds, and could potentially be used in the field of ear rot management caused by *Stenocarpella* spp. due to their stability at different temperatures and pH conditions.

In a *B. amyloliquefaciens* strain was reported that Iturin A is the principal compound with antifungal activity that inhibits different postharvest fungal pathogens. The inhibitory effect was observed in abnormal conidial germination and germ tube development when conidia were treated when different lipopeptides extracts [38]. The thermostable filtrates obtained in this work are not cytotoxic and have the ability to cause abnormal germination of conidia.

The method most used for the control of *S. maydis* and *S. macrospora* in the field is crop rotation, however this practice requires suspending corn production by several years with the objective of decreasing the spore inoculum in the soil. On the other hand, the utilization of commercial hybrids has rarely been effective and losses are very important during harvest and post-harvest. In this last aspect, farm animals rejected the stored grains infected by *Stenocarpella* and their nutritional value is diminished [8]. Even more, *S. maydis* produces the diplodiatoxin a mycotoxin associated with different ailments in domestic ruminants, ducks, chickens, and horses [4–6]. The biological control of ear rot has scarcely been studied, with one report of isolation and utilization of actinomycetes at the experimental level, focused on strains of the *Streptomyces* genus with the potential to be used in the field [12].

## 3. Experimental Section

### 3.1. Fungal strains and Growth Conditions

The strains used in this study were *S. maydis* 2 and *S. macrospora* 533-4 kindly provided by Dr. Dan Jeffers from the Centro Internacional de Mejoramiento de Maíz y Trigo (CIMMYT), Mexico.

The fungal strains were grown in Petri plates containing potato dextrose agar medium (PDA) and YEPD solid medium (1% yeast extract, 2% peptone, 2% dextrose and 2% agar). The plates were maintained at 28 °C for 8 days, and then kept at −4 °C.

The following media were used to grow the bacterial strains, trypticasein soy agar medium (TSA), PDA medium at 28 °C, Kings medium B and potato dextrose broth medium (PDB) on a rotary shaker at 200 rpm at 28 °C for 20 h, and each strain was maintained in 25% glycerol at −80 °C.

### 3.2. Isolation and Selection of Bacteria with Antifungal Activity

Bacteria were isolated from rhizospheric soil samples obtained from corn crops in the state of Hidalgo, located in the central part of Mexico. Suspensions with 10 g of the sampled soil and 90 mL of sterile distilled water were prepared; each suspension was diluted from 10^−1^ until 10^−8^. From these dilutions, 0.1 mL were inoculated in plates with TSA and Kings medium B and incubated at 28 °C. The plates were observed every 12 h for 72 h. Colonies with different characteristics were selected and preserved in TSA.

All isolated bacteria were tested for antifungal activity as follows: a 1-cm^2^ fungal plug (*S. maydis* or *S. macrospora)* was inoculated in the center of a plate with PDA, each isolated bacterium was sown with a sterile stick at a distance of 2.5 cm from the fungus. The plates were then incubated at 28 °C for 72 h and verified every 12 h. The strains with capacity to inhibit proliferation of the fungus were selected and preserved. For an initial classification of the bacterial strains, was necessary to perform a morphological characterization of the colony and Gram staining [39].

### 3.3. Identification of the Bacterial Strains with Antifungal Activity

The bacterial strains were identified by molecular methods by the amplification and sequencing of the 16S rRNA using the following universal primers: 8F 5′-AGAGTTTGATCCTGGCTCAG-3′ and 1492R 5′-GTTACCTTGTTACGACTT-3′. The PCR conditions were initial denaturalization for 5 min at 95 °C, 30 cycles of 60 s at 95 °C, 60 s at 54°C, 90 s at 72 °C and the final extension for 10 min at 72 °C. The PCR products were cleaned with the QIAquick PCR purification kit (Qiagen, USA) following the manufacturer instructions. The PCR-products were sequenced in both directions in an Applied Biosystems model 3730XL automated DNA sequencing system. Both strands of DNA were compared and edited manually. Multiple alignments of sequences were carried out with Clustal X [40]. A maximum likelihood (ML) analysis was done in PAUP*(v.4.0b10) [41] using a heuristic search strategy with tree bisection-reconnection: ACCTRAN and 10 random-taxon-addition replicates, starting from a random tree. Confidence at each node was assessed using a bootstrap analysis based on 5000 pseudoreplicates with 10 random-taxon-addition replicates per pseudoreplicate.

### 3.4. Collection and test of Antifungal Activity of Extracellular Filtrates

To obtain extracellular filtrates with antifungal activity, samples were collected in logarithmic phase and at the beginning and end stationary phase of the growth of each isolated bacterium with antifungal activity, cultured in PDB medium, at 28 °C, at 180 rpm. The samples were centrifuged at 8000 rpm and the supernatants (extracellular fraction) were sterilized by filtration, using a 0.22-μm pore diameter membrane, and then 200 μL to the filtrate was added in small bottomless tubes placed on PDA plates at a distance of 2.5 cm from the center to where the fungi were inoculated. The plates were incubated at 28 °C for 120 h and verified every 12 h. The filtrates with antifungal activity were those that inhibited the development of the fungi.

### 3.5. Evaluation of the pH and the Temperature in the Stability of Extracellular Filtrates with Antifungal Activity and Determination of Their Cytotoxicity

To evaluate the effect of the pH and the temperature in the stability of the extracellular filtrates, they were incubated during 1, 7, 15, and 30 days at different pH conditions (3.6, 7, and 10 pH values) and different temperatures (−20, 4, 18, 37, 50 °C); in the first case, 500 μL of the filtrate was mixed with 500 μL of different buffers (0.5 M citrate buffer, pH 3.6; 0.5 M phosphate buffer, pH 7; 0.5 M Tris-HCl buffer, pH 10), as a control, 500 μL of PDB was mixed with the different buffers. One sample of these filtrates was sterilized in an autoclave. All filtrates were tested to determine their antifungal activity, as described previously. Results are reported as percentage antifungal activity. The brine shrimp lethality test was used to evaluated the cytotoxicity of those filtrates that were thermostable according at the procedures described by Solis *et al*. [42].

### 3.6. Effect of the Extracellular Filtrates with the Antifungal Activity against Conidia Germination

The conidia were obtained from pycnidia, induced by growing the fungi for 25 days at 28 °C with periods of 12 h light and 12 h darkness in 200 wood toothpicks were placed in beakers of 100 mL with 25 mL of PDB diluted 1:2 with distilled water. The pycnidia were collected in sterile water and were then vigorously shaken to liberated the conidia. This solution was diluted until 10^−5^ and 0.1 mL were mixed with 300 μL of the each thermostable extracellular filtrate and inoculated in YEPD plates, then were incubated for 96 h at 28 °C. Each plate was observed in an optical microscope.

## 4. Conclusions

The bacterial strains that are described in this work have potential as biological control agents. The strains of *B. subtilis* are the most promising due to their ability to produce endospores that can remain in the soil during long periods and can be produced easily at an industrial level with a low cost [43]. This last is important for product preparations in which it is possible to incorporate antifungal filtrates stable to heat and pH changes to control the disease in its early state and for the production of spores to be inoculated into the soil to prevent future infections in the cultivars.

The results obtained will be used for the design of new environmentally friendly methodologies for the control of *S. maydis* and *S. macrospora* without using chemical compounds that contaminate agricultural soils.

## Figures and Tables

**Table 1:** Antifungal activity on *S. maydis* and *S. macrospora* of extracts obtained from the isolated bacteria in different growth phases.

Strain bacteria*	Percent of inhibition (%)					
	Logarithmic phase		Early stationary phase		Late stationary phase	

	*S. maydis*	*S. macrospora*	*S. maydis*	*S. macrospora*	*S. maydis*	*S. macrospora*
*Pseudomonas* spp. 11	**73.0 ± 0.04**	**71.1 ± 0.06**	**73.8 ± 0.06**	**81.0 ± 0.06**	**76.1 ± 0.06**	**81.2 ± 0.08**
*B. subtilis* 13	17.8 ± 0.04	35.0 ± 0.08	76.6 ± 0.04	77.4 ± 0.07	**79.8 ± 0.06**	**79.8 ± 0.05**
*P. fluorescens* 16	18.0 ± 0.06	19.0 ± 0.04	**55.2 ± 0.06**	5.1 ± 0.06	24.2 ± 0.04	4.0 ± 0.04
*P. agglomerans* 19	0.0 ± 0.00	0.0 ± 0.00	1.0 ± 0.04	7.2 ± 0.05	1.0 ± 0.03	16.7 ± 0.04
*Pseudomonas* spp. 21	0.0 ± 0.00	0.0 ± 0.00	1.0 ± 0.02	10.4 ± 0.04	1.0 ± 0.03	3.8 ± 0.06
*B. subtilis* 35	22.8 ± 0.05	59.6 ± 0.04	73.4 ± 0.04	73.3 ± 0.03	**76.6 ± 0.04**	**81.9 ± 0.03**
*B. subtilis* 55	10.4 ± 0.04	10.3 ± 0.05	**88.3 ± 0.01**	**76.9 ± 0.03**	79.3 ± 0.04	74.3 ± 0.04
*B. subtilis* 135	9.6 ± 0.04	16.6 ± 0.05	88.4 ± 0.05	70.8 ± 0.04	**96.5 ± 0.02**	**84.3 ± 0.04**
*Pseudomonas* spp. 156	2.0 ± 0.03	0.0 ± 0.00	5.0 ± 0.06	2.4 ± 0.07	5.0 ± 0.06	0.8 ± 0.01
*B. subtilis* 160	2.2 ± 0.06	34.3 ± 0.04	34.4 ± 0.03	46.1 ± 0.06	**89.0 ± 0.05**	**80.0 ± 0.06**

* The bacterial strains were cultured in PDB medium, as described in Materials and Methods. The assays were performed in quadruplicate.
